# Incidental Finding of Invasive Ductal Carcinoma on Mastectomy in the Case of Lymphocytic Mastitis: A Case Report

**DOI:** 10.7759/cureus.44545

**Published:** 2023-09-01

**Authors:** Sweta D Bahadure, Obaid Noman, Neha Jaiswal, Gulshan Bandre, Anil Akulwar

**Affiliations:** 1 Department of Pathology, Datta Meghe Medical College, Datta Meghe Institute of Higher Education and Research (Deemed to be University), Wardha, IND; 2 Department of Microbiology, Jawaharlal Nehru Medical College, Datta Meghe Institute of Higher Education and Research (Deemed to be University), Wardha, IND; 3 Department of Surgery, Datta Meghe Medical College, Datta Meghe Institute of Higher Education and Research (Deemed to be University), Wardha, IND

**Keywords:** case report, biopsy, mastectomy, histopathology, infiltrating ductal carcinoma, lymphocytic mastitis

## Abstract

A rare inflammatory breast disorder called lymphocytic mastitis is characterized by lymphocyte infiltrates in the mammary parenchyma. Due to their rarity, incidental observations of invasive ductal carcinoma in lymphocytic mastitis present diagnostic and management challenges.

We present a case of a 52-year-old female with a history of painfully swollen breasts for three months who underwent a core needle biopsy, consistent with lymphocytic mastitis on histopathology. Due to persistent and worsening symptoms, a mastectomy was performed. During the examination, an incidental finding of infiltrating ductal carcinoma was identified in the mastectomy specimen. This unexpected discovery led to further investigations and altered the patient's treatment plan. The detection of invasive ductal carcinoma in the presence of lymphocytic mastitis highlights the importance of continuous surveillance and thorough examination. In the circumstances of lymphocytic mastitis, it is vital to take the likelihood of concurrent malignancy into account, especially when symptoms persist or reappear after appropriate management. This case report seeks to raise awareness among physicians of this exceptional association and drive further research that will explain its pathophysiology while enhancing management strategies.

## Introduction

Lymphocytic mastitis is commonly seen in patients with Type I diabetes mellitus or other autoimmune diseases. Hence, it is also termed diabetic mastopathy and diabetic fibrous breast disease [1]. Clinically and radiologically, the lesion may mimic breast carcinoma. While the condition is relatively uncommon, it can cause significant discomfort and pose diagnostic challenges [2]. This case report aims to describe a case diagnosed as lymphocytic mastitis on imaging and biopsy and to highlight the findings of an infiltrating ductal carcinoma (IDC) discovered during a mastectomy procedure executed due to the persistence of symptoms.

This case report emphasizes the significance of taking malignancy into account when treating lymphocytic mastitis, especially when symptoms persist or get worse over time. It also highlights the importance of carefully inspecting the removed breast tissue after surgery to find any related cancers. For an accurate diagnosis and the best course of treatment, prompt action, such as a mastectomy, is essential [3].

## Case presentation

We present the case of a 52-year-old female with a three-month history of a painful, swollen right breast. She complained of diffuse breast tenderness, warmth, and a rash on the affected breast. There was no nipple discharge or palpable breast mass. She had type 2 diabetes mellitus for the last 10 years and was on insulin injections for it. The patient denied any previous history of breast disease or family history of breast cancer. A thorough examination of both breasts was performed, noting the presence of an erythematous rash, tenderness, and warmth in the right breast. Routine lab investigations and mammography were advised, and conservative management was given.

Diagnostic evaluation

**Figure 1:**
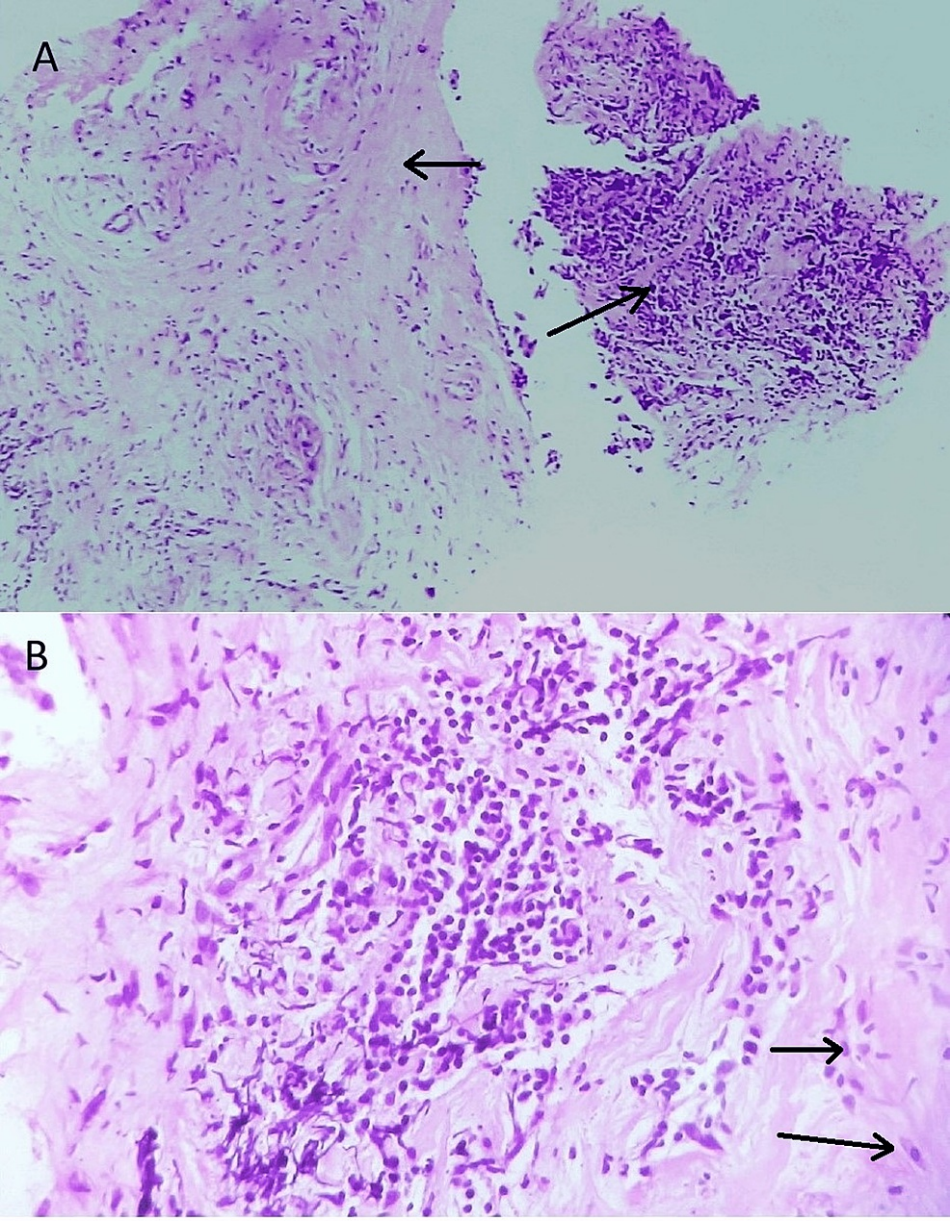
Lymphocytic mastitis showing A. lymphocytic inflammatory infiltrate and dense fibrosis (H&E 40x); B. epitheloid stromal myofibroblast (H&E 100x) H&E (40x): hematoxylin and eosin, scanner view; H&E (100x): hematoxylin and eosin, low power view

Laboratory investigation revealed an increase in fasting blood sugar (123 mg/dl), postprandial blood sugar (162 mg/dl), elevated ESR (48 mm/hour), and elevated C-reactive protein (12 mg/L). A mammogram demonstrated diffuse edema and increased density of the right breast parenchyma. The patient’s symptoms persisted and worsened since the initial visit, despite conservative management. To determine the underlying cause of the symptoms and assess for malignancy, a core needle biopsy of the affected breast tissue was performed. The biopsy sample was sent for histopathological analysis, which revealed dense fibrosis with periductal, perilobular, and perivascular lymphocytic inflammatory infiltrates and the collection of epitheloid stromal myofibroblasts (Figure 1).

No malignant cells were identified. The patient was managed conservatively with non-steroidal anti-inflammatory drugs (NSAIDs) and supportive measures, including warm compresses and pain management. However, despite these measures, the patient’s symptoms persisted and worsened over time. Additional ultrasonography (USG) was performed, but the findings were inconclusive. Due to the intractable pain and concern for underlying malignancy, the decision was made to proceed with a modified radical mastectomy.

**Figure 2:**
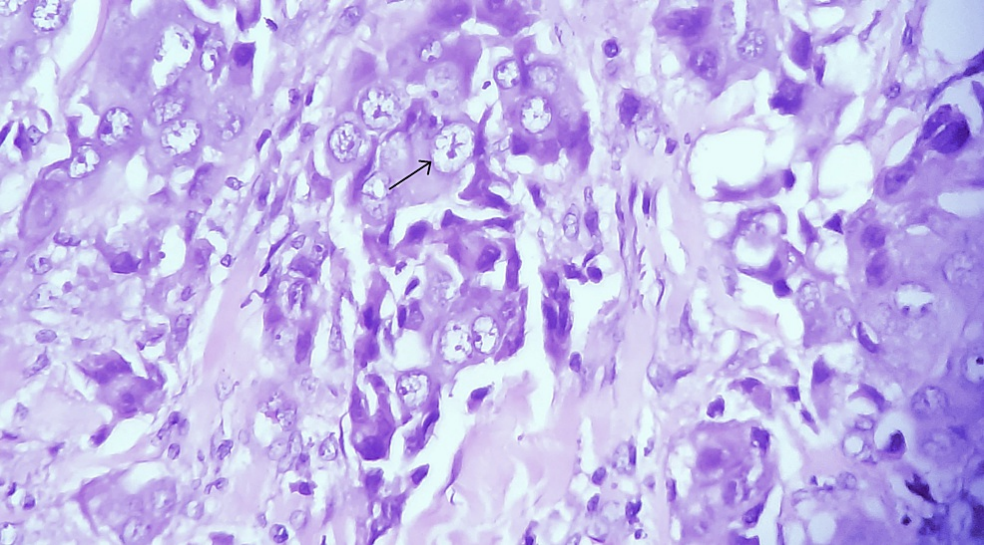
Infiltrating ductal carcinoma showing tumor cells with eosinophilic cytoplasm and pleomorphic nuclei with prominent and multiple nucleoli (H&E 400x) H&E (400X): hematoxylin and eosin, high-power view

**Figure 3:**
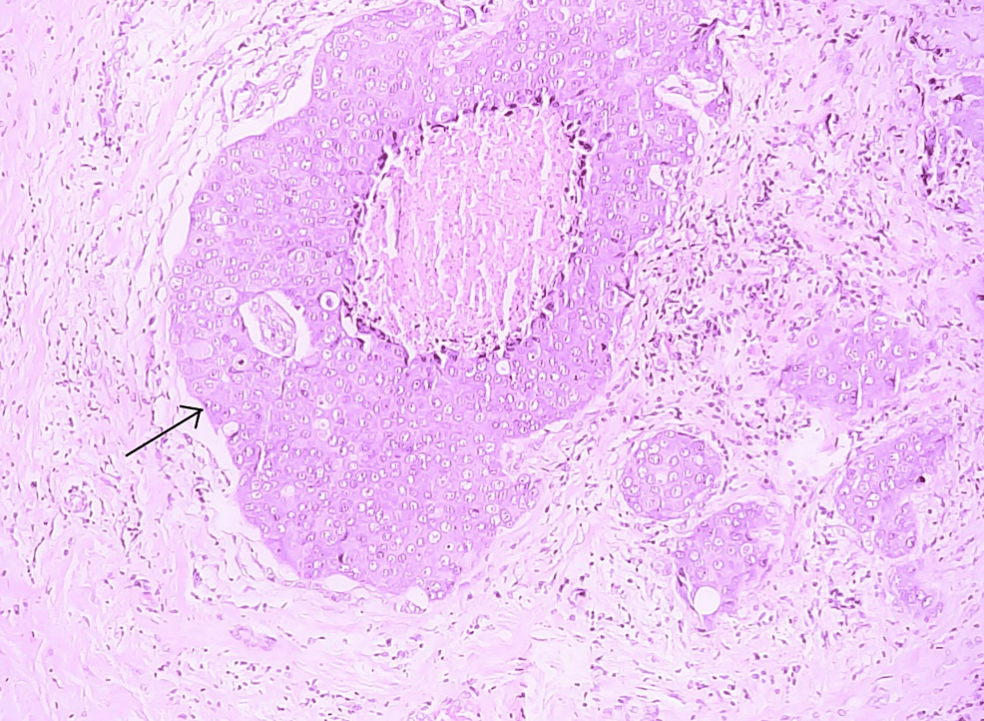
Infiltrating ductal carcinoma showing comedo necrosis (H&E 100x) H&E (100X): hematoxylin and eosin, low-power view

A mastectomy was performed, and the specimen was sent for histopathological examination. During the processing of a specimen, a careful examination of the excised breast tissue revealed a 1.2 cm mass within a dilated duct, suspicious of intraductal carcinoma. The mass was located in close proximity to an area of prominent lymphocytic infiltration. The surrounding breast tissue showed evidence of chronic inflammation and fibrosis. Microscopic examination of that suspicious mass confirmed the presence of IDC. The tumor was characterized by atypical ductal epithelial cells with high nuclear grade and comedo necrosis (Figures 2, 3).

Axillary lymph node dissection showed no evidence of metastasis. An immunohistochemical study revealed estrogen receptor (ER) positivity, progesterone receptor (PR) negativity, and human epidermal growth factor (HER 2) negativity.

Follow-up and outcome

Postoperatively, the patient had an uneventful recovery. She was referred to an oncologist for further evaluation and management. The patient underwent adjuvant chemotherapy followed by radiation therapy. Additionally, she received hormonal therapy due to ER positivity on immunohistochemistry. Regular surveillance with imaging and clinical examinations was initiated. At the six-month follow-up, the patient remained disease-free with no evidence of recurrence.

## Discussion

Lymphocytic mastitis, also known as diabetic mastopathy and sclerosing lymphocytic lobulitis, is a very uncommon breast ailment [4]. Lymphocytic infiltration and inflammation of the breast tissue, keloidal type of fibrosis, and the presence of epitheloid myofibroblast are its defining features [5]. Numerous studies have linked it to long-term type I diabetes and other autoimmune diseases like Hashimoto thyroiditis, pernicious anemia, rheumatoid arthritis, Sjogren syndrome, systemic lupus erythematosus, etc. [6-7]. It can also be seen in type II diabetes patients and in people who are not diabetic [8]. Some theories propose an autoimmune reaction against breast epithelial antigens, despite the fact that the precise etiology is unknown [9]. Other theories suggest that hyperglycemia causes stromal matrix expansion and the accumulation of glycosylation end products, which give rise to an inflammatory response in B cells [10]. Few suggest an immunologic response to exogenous insulin [11].

Breast soreness, swelling, warmth, and erythema are common symptoms, and they might mimic other inflammatory diseases like mastitis or abscess. In cases where symptoms are chronic or get worse, it is crucial to take the possibility of cancer into account [2]. The patient in this case report had early diagnostic testing, including a core needle biopsy, which showed significant lymphocytic infiltration that was consistent with lymphocytic mastitis. At that time, no malignant cells had been discovered. The patient was managed conservatively with NSAIDs and supportive measures but experienced persistent and worsening symptoms, leading to the decision to perform a mastectomy.

During the mastectomy procedure, the presence of an IDC was discovered within a dilated duct located in close proximity to the area of lymphocytic mastitis. This finding emphasizes the significance of careful examination of breast tissue during grossing. The diagnosis of IDC, which was supported by atypical ductal epithelial cells with a high nuclear grade and comedo necrosis, was made after a histopathological examination. The IDC's close proximity to lymphocytic mastitis lends further credence to the idea that there might be a connection between the two diseases. Although extremely uncommon, breast cancer has been documented to develop from lymphocytic mastitis [12-13]. The underlying processes or danger elements in this shift are still unknown [1,4]. According to some research, cancer may develop as a result of persistent inflammation, frequent cycles of damage and repair, and other factors. The inflammatory milieu brought on by lymphocytic mastitis may be a factor in the cellular and genetic modifications that favor the growth of IDC. To understand the pathophysiology and find relevant biomarkers for foretelling this transition, more investigation is required [14]. Given that the IDC was discovered at an early stage in this case, the patient's prognosis was deemed positive. The likelihood of a successful course of therapy and favorable long-term outcomes for breast cancer is greatly increased by early detection and management. For additional assessment and management of the IDC, the patient was referred to an expert in oncology.

In-depth knowledge of the risk factors, pathophysiological mechanisms, methodologies for diagnosis, and therapeutic approaches is necessary to ascertain the relationship between lymphocytic mastitis and IDC. The aforementioned information will promote early diagnosis, improve patient outcomes, and increase diagnostic accuracy.

## Conclusions

The unanticipated discovery of infiltrating ductal carcinoma in a case of lymphocytic mastitis emphasizes the value of thorough assessment and the need to take cancer into account in patients who present with prolonged or unusual clinical symptoms. In order to ensure prompt diagnosis and effective management, medical personnel should be cognizant of the unusual association between lymphocytic mastitis and IDC. It is necessary to further comprehend the relationship between lymphocytic mastitis and IDC, establish standardized criteria for the diagnosis, and conduct additional research and clinical trials.
